# Pattern of Regional Cerebral Metabolism before Onset of Clinically Evident Lewy Body Disease Differentially Predicts Future Lewy Body vs. Alzheimer’s Symptom Progression among Patients with Parietotemporal Hypometabolism at Baseline

**DOI:** 10.1002/alz70861_108853

**Published:** 2025-12-23

**Authors:** Natalie Mahgerefteh, Chana Fink, Mahbod Jafarvand, Karen Ke, Daniel H Silverman

**Affiliations:** ^1^ University of California, Los Angeles, Los Angeles, CA USA

## Abstract

**Background:**

Regional cerebral metabolic patterns can distinguish demented patients with Alzheimer's disease (AD) from those with Lewy bodies (LB) during life, correlating with clinical diagnoses that are subsequently confirmed by autopsy. While clinical LB is characterized by ≥2 core symptoms (visual hallucinations, REM behavior disorder, cognitive fluctuations, parkinsonism), PET imaging may detect LB‐characteristic metabolic patterns years before that diagnosis is made clinically. This study aimed to more fully characterize the predictive value of metabolic imaging with respect to subsequent emergence of LB‐related symptoms.

**Method:**

Sixty patients who underwent FDG‐PET at our institution in 2018‐2022 were clinically followed through March 2025. Metabolic patterns were visually categorized as LB‐type (*n* =30) or AD‐type (*n* =30) based on visual analysis by nuclear medicine physicians blinded to clinical history. Ratio of activity in bilateral posterior cingulate to associative visual cortical standardized volumes of interest (PCC/AVC) were also calculated for each patient, and correlations with symptom progression were examined at 1‐year, 2‐year, and final follow‐up.

**Result:**

PCC/AVC ratio was significantly higher in the LB‐type group (av. 1.19 vs. 1.04; *p* <0.0001), with high ratios (>1.12) predicting greater symptom progression (*p* =0.0275). Both groups had identical follow‐up durations (av. 31 months, *p* =0.8). Patients with LB‐type metabolism showed greater increase in number of LB core (+0.87 vs. +0.03, *p* =0.00009) and total (+2.23 vs. +0.57; *p* =0.00112) symptoms between baseline and final evaluations. At baseline, below‐median LB‐type and above‐median AD‐type groups had similar symptom counts, yet the below‐median LB‐type group developed more core symptoms during follow‐up (*p* =0.0012). By 1 year, patients with LB‐type metabolism were more likely to have developed each of the core symptoms: hallucinations (*p* <0.0001), REM behavior disorder (*p* =0.0024), fluctuations (*p* =0.025), and parkinsonism (*p* =0.030). By 2 years, LB‐type patients were more likely to have developed ≥2 core symptoms (*p* =0.00077).

**Conclusion:**

Patients clinically non‐LB but with LB‐type cerebral metabolism at baseline developed more clinical LB features by one year and were more likely to suffer from ≥2 core LB symptoms by two years, including when matched for baseline number of core symptoms, while PCC/AVC ratios measured at the individual level correlated with degree of LB‐symptom progression over subsequent years.

